# Novel applications of machine learning and computational neuroscience models to neuroimaging in Parkinson’s disease and related disorders

**DOI:** 10.3389/fnagi.2026.1786423

**Published:** 2026-03-09

**Authors:** Lydia Chougar, Andrew Vo, Stéphane Lehéricy, Alain Dagher

**Affiliations:** 1 The Neuro - Montreal Neurological Institute and Hospital, McGill University, Montreal, QC, Canada; 2 Sorbonne Université, Institut du Cerveau - Paris Brain Institute - ICM, AP-HP, CNRS, Inserm, Hôpital de la Pitié Salpêtrière, Department of Neuroradiology, Paris, France

**Keywords:** atypical parkinsonism, computational models, diagnosis, machine learning, MRI, Parkinson’s disease, pathophysiology, subtyping

## Abstract

**Purpose of review:**

Parkinsonian syndromes are a heterogeneous group of neurodegenerative diseases that pose challenges in early diagnosis, differentiation, and pathophysiological understanding. The objective of this review is to summarize recent contributions of computational models combined with neuroimaging data to the differential diagnosis of Parkinsonian syndromes, disease subtyping, and understanding of disease processes.

**Recent findings:**

Using machine learning algorithms trained with MRI features, diagnostic accuracies above 90% have been achieved for distinguishing patients with Parkinson’s disease from healthy controls and for the differential diagnosis of Parkinsonian syndromes. Computational models, such as hierarchical cluster analysis and Subtype and Stage Inference (SuStaIn), have enabled the identification of distinct disease subtypes within Parkinson’s disease based on imaging-derived brain features. Network models based on structural and functional connectomes have revealed that disease spread in Parkinson’s disease is primarily driven by global connectivity. Additionally, local brain characteristics such as gene expression, cellular composition, and neuroreceptor profiles may contribute to selective vulnerabilities.

**Summary:**

Computational approaches enhance the diagnosis of Parkinsonian syndromes, particularly in the early stages, and refine the characterization of disease subtypes, benefiting clinicians, especially in non-expert centers. Such applications hold significant potential for enabling more personalized management and selecting appropriate candidates for clinical trials. Furthermore, a deeper understanding of pathophysiology supports the development of disease-specific therapies.

## Highlights

- Machine learning methods combined with neuroimaging data can accurately differentiate Parkinsonian patients from healthy subjects and discriminate among Parkinsonian syndromes
- Computational models help distinguish disease subtypes within PD
- Computational models are powerful tools for exploring the underlying pathophysiology within PD and their subtypes

## 1 Introduction

**Figure 1:**
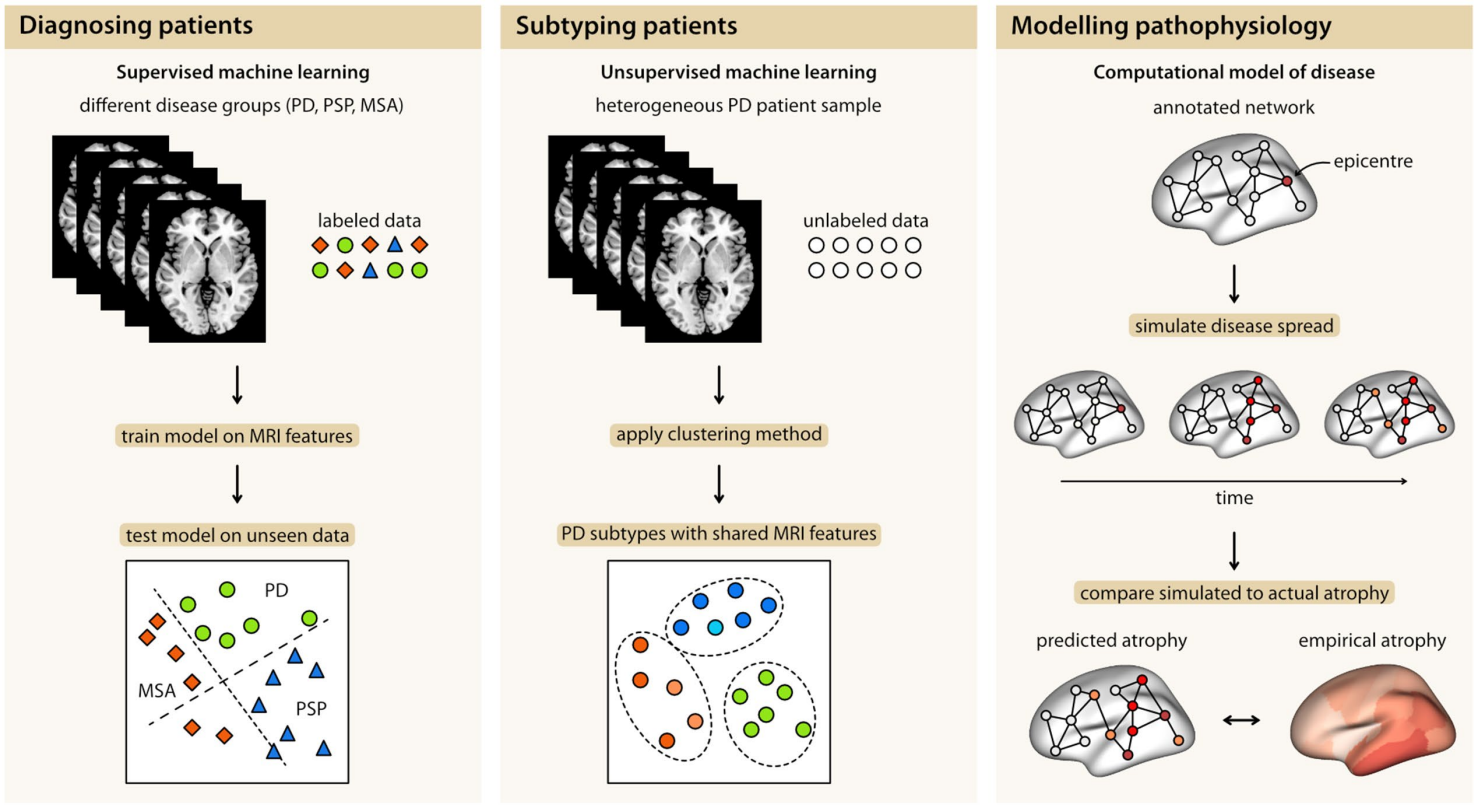
The three main contributions of computational models in combination with MRI data: Diagnosing Parkinsonian syndromes, identification of disease subtypes within Parkinson’s disease, and modeling disease pathophysiology. MSA, multiple system atrophy; PD, Parkinson’s disease; PSP, progressive supranuclear palsy.

In recent years, the field of Parkinson’s disease (PD) and Parkinsonism has experienced a substantial increase in the availability of clinical, imaging, and omics data, particularly from open-source multicenter cohorts. This abundance of data allows for the investigation of Parkinsonism across multiple modalities in well-powered samples. However, it also adds complexity to the analysis, which can be addressed using computational approaches. Machine learning (ML) approaches aim to uncover statistical patterns in high-dimensional data, such as neuroimaging, and are employed to develop diagnostic or prognostic biomarkers, whereas computational methods encompass a broad range of techniques that utilize algorithms to model disease processes. In this review, we will focus on recent studies and provide an overview of how computational models combined with neuroimaging data can be used to differentiate patients with PD from healthy subjects and other Parkinsonian syndromes, distinguish subtypes within PD, and explore the underlying pathophysiology of PD and its subtypes (Figure 1). We will conclude the review by outlining key challenges and promising developments in ML and computational science that pave the way toward clinical translation.

## 2 Diagnosing parkinsonism using machine learning approaches

ML has been extensively used in research for diagnostic classification of neurodegenerative diseases in recent years, especially in the context of Parkinsonism. ML learns from data, identifies patterns, and classifies data, enabling inference at the individual level (Mei et al., 2021; Chougar et al., 2021). Following data preparation and feature extraction, the development of an ML model involves three steps: first, training the algorithm; second, validating its performance; finally, testing its predictive accuracy either on an independent dataset or using cross-validation when the sample is limited. ML can be either supervised (i.e., trained with pre-labeled data) or unsupervised (i.e., trained with unlabeled data and entirely data-driven). Supervised ML encompasses techniques such as support vector machine (SVM), artificial neural networks (ANNs), and random forest, while unsupervised ML includes methods such as clustering. Deep learning is a particular type of ML that uses a model made of a very large set of artificial neurons organized into layers (Burgos and Colliot, 2020). A wide range of imaging modalities, such as single photon emission computed tomography (SPECT) (Choi et al., 2017; Oliveira et al., 2018; Taylor et al., 2018; Ortiz et al., 2019; Magesh et al., 2020), positron emission tomography (PET) (Zhao et al., 2019; Wu et al., 2019; Shen et al., 2019; Glaab et al., 2019), and structural (Adeli et al., 2016; Kamagata et al., 2016; Singh et al., 2018; Amoroso et al., 2018; Gaurav et al., 2022; Prasuhn et al., 2020; Chakraborty et al., 2020; Solana-Lavalle and Rosas-Romero, 2021) and functional MRI (Baggio et al., 2019), have been used to train ML. These applications have been applied to two main tasks: (i) differentiating patients with PD from healthy controls (HC) and (ii) differential diagnosis of Parkinsonism (Mei et al., 2021; Chougar et al., 2021). We provide an overview of both applications in the following sections.

### 2.1 Diagnosis of Parkinson’s disease

Some studies have used ML combined with imaging features to discriminate PD patients from HCs. Notably, a study extracted voxel-based morphometric features in a large cohort of 348 patients with PD and 159 HCs from the Parkinson’s Progression Markers Initiative (PPMI) study, which were used to train several supervised algorithms (including k-nearest neighbors, support vector machine, random forest, naive Bayes, and logistic regression) with a tenfold cross-validation, without an independent testing set. High predictive performance was obtained with accuracies ranging from 0.93 to 0.99 (Solana-Lavalle and Rosas-Romero, 2021). Similarly, using a convolutional neural network (CNN) model trained with morphometric features in PPMI, another study obtained a 95.3% accuracy. The class activation maps highlighted the substantia nigra as the main contributor to classification, adding a pathophysiological value to the findings (Chakraborty et al., 2020). Focusing on the substantia nigra using neuromelanin-sensitive imaging as input for a CNN model, another study achieved an 80% accuracy for the discrimination of PD versus HC (Gaurav et al., 2022).

Diffusion imaging studies have repeatedly reported brain microstructural alterations in PD, especially in the substantia nigra (Burciu et al., 2016; Burciu et al., 2017; Atkinson-Clement et al., 2017; Chougar et al., 2020). More recently, Free Water (FW) diffusion imaging has been developed to estimate the fractional volume of freely diffusing water molecules within a voxel using a bitensor model. FW is expected to increase with neurodegeneration (Golub, 2018). Therefore, approaches combining diffusion data with ML methods are expected to have great discriminative power. However, a classification model based on diffusion imaging with ROI-based and whole-brain analysis approaches combined with SVM or multiple-kernel learning failed to discriminate PD patients from HCs (accuracies: 48–50%), even when focusing on the substantia nigra (Prasuhn et al., 2020). More studies are needed to investigate the ability of ML models trained with diffusion data to diagnose PD.

### 2.2 Differential diagnosis of parkinsonism

While distinguishing PD patients from HC has been a recurrent research question, it is less relevant in clinical practice, where patients often present with overlapping symptoms that span multiple diagnostic categories, including degenerative Parkinsonism, Essential tremor, neuroleptic-induced Parkinsonism, or normal pressure hydrocephalus. The true challenge lies in differentiating PD from these conditions, or from atypical Parkinsonian syndromes such as progressive supranuclear palsy (PSP) or multiple system atrophy (MSA). Neuroimaging shows disease-specific patterns that, when combined with ML algorithms, enable diagnostic classification at the individual level. Such an approach holds the potential to enhance the diagnosis of Parkinsonism, especially at early stages when international clinical criteria may have lower accuracy (Table 1; Scherfler et al., 2016; Chougar et al., 2024).

**Table 1:** Summary of the main studies using machine learning algorithms trained on MRI features to differentiate parkinsonian syndromes.

Study	Population					Validation approach	Features	Algorithm	Performances
	*n*	HC	PD	MSA	PSP				
Huppertz et al. (2016)	464	73	204	81	106	Cross-validation	Volumetry	SVM linear and RBF	Accuracy >80%
Scherfler et al. (2016)	110		40	40	30	External test set	Volumetry	Decision tree	PSP vs. PD, MSA vs. PD: 97%
Du et al. (2017)	106	36	35	16	19	Cross-validation	Diffusion metrics, R2* relaxometry	Elastic-net	AUC > 0.88
Morisi et al. (2018)	85		47	16	22	Cross-validation	Volumetry, diffusion metrics, spectroscopy	SVM	Binary classifications: AUC 0.94–0.98; Three class classification: 91%
Péran et al. (2018)	81	26	26	29	–	Cross-validation	VBM, diffusion metrics, R2* relaxometry	Logistic regression, non-supervised machine learning	AUC > 0.95
Shinde et al. (2019)	–	35	45	20		Cross-validation and external test set	Neuromelanin imaging: substantia nigra signal and volume; Texture parameters	CNN, gradient boosting	Accuracy 80%
Kiryu et al. (2019)	419	141	125	54	98	Held-out validation set (internal validation set)	Morphometry; single midsagittal T1-weighted MRI	CNN	AUC 0.94–0.98
Abos et al. (2019)	150	54	65	31	–	Cross-validation	Structural connectivity; FA and MD	SVM with leave-one-out-cross validation	PD vs. MSA and HC vs. MSA: AUC 0.78
Baggio et al. (2019)	151	59	62	30	–	Cross-validation	Functional connectivity	SVM	PD vs. MSA: AUC 0.77
Archer et al. (2019)	1,002	278	511	84	129	External test set	Free water, free water corrected fractional anisotropy	SVM	AUC > 0.89
Chougar et al. (2020)	322	94	119	58	51	Cross-validation and external test set	Volumetry, diffusion metrics	Logistic regression, SVM, Random forest	Binary classifications: 77–98%; Three class classification: 77%
Tsai et al. (2023)	625	219	286	51	69	Cross-validation and external test set	Diffusion metrics	SVM, DFA	PD vs. HC: AUC 87%; PD vs. APS: 83%
Vaillancourt et al. (2025)	645	–	310	151	184	External test set	Free water, free water corrected fractional anisotropy	SVM	AUC > 0.98
Mattia et al. (2025)	156	–	64	92	–	Cross-validation	Gray matter density and MD	CNN	Accuracy 0.84

APS, atypical parkinsonian syndrome; AUC, area under the ROC curve; CNN, convolutional neural network; DFA, discriminant function analysis; FA, fractional anisotropy; HC, healthy control; MD, mean diffusivity; MSA, multiple system atrophy; PD, Parkinson’s disease; PSP, progressive supranuclear palsy; R2*, transverse relaxation rate; Se, sensitivity; SN, substantia nigra; Sp, specificity; SUV, standardized uptake value; SVM, support-vector machine; TSPO, Translocator protein.

Several studies have used brain volumetric data extracted from T1-weighted images to train ML algorithms on large cohorts. Overall, high accuracies were achieved, ranging between 77 and 98% (Gaurav et al., 2022; Scherfler et al., 2016; Chougar et al., 2024; Huppertz et al., 2016; Morisi et al., 2018; Péran et al., 2018). Classification performance was also high when using a CNN model trained on a single T1-weighted midsagittal slice to distinguish PD, PSP, and MSA (Kiryu et al., 2019).

Diffusion-weighted imaging has also been shown to be useful for differentiating Parkinsonian syndromes. A notable multicenter study investigated the use of FW and FW-corrected fractional anisotropy measured in 60 brain regions in combination with a linear SVM. It included the largest cohort to date, comprising 1,002 subjects: 278 HC, 511 PD, 129 PSP, and 84 MSA. High accuracy was achieved for all classifications (AUC > 89%) using the diffusion metrics alone or when combined with UPDRS-III scores (Archer et al., 2019). More recently, the same group replicated these results in a new prospective cohort and validated them against neuropathology in 93.8% of autopsy cases (Vaillancourt et al., 2025).

The potential for clinical translation of such findings was investigated in another study that replicated an ML-based diagnostic classification, originally designed in research, in a clinical sample. Using a cohort of 322 subjects (94 HC, 119 PD, 51 PSP, and 58 MSA) split into a training cohort (*n* = 179) and a replication cohort (*n* = 143), the authors extracted volumes and diffusion metrics in 13 regions of interest. Four supervised ML algorithms, including logistic regression, SVM, and random forest, were trained. High performances were obtained in the test cohort for most binary classification tasks (balanced accuracies: 77–98%). Adding diffusion features did not improve the performance compared to volumetry only. These findings hold promise that AI-based diagnostic approaches could be transferred into clinical routine (Chougar et al., 2020).

The added value of a fully automated, data-driven, whole-brain pipeline using a CNN model trained with gray matter and mean diffusivity maps was recently explored in a multicenter cohort of 64 PD and 92 MSA patients. High performances were achieved (88.8% accuracy), with a relatively short computational time per subject, including both image processing and prediction. Interestingly, model interpretability was investigated by analyzing misclassified patients and providing a visual interpretation of the most activated regions in CNN predictions, thereby enhancing the clinical plausibility of the approach (Mattia et al., 2025).

This section highlights the growing evidence that ML approaches using imaging data show promise for improving the diagnosis of Parkinsonian syndromes and supporting early detection.

## 3 Disease subtyping and stratification

PD is highly heterogeneous and can manifest as significantly different profiles of clinical symptoms, disease progression, and treatment responses from one patient to another (Foltynie et al., 2002). This aspect of PD poses substantial challenges for diagnosis and symptom management. Identifying disease subtypes based on imaging-derived brain features could allow for more patient-tailored therapeutics and the selection of appropriate candidates for clinical trials.

One approach to disease subtyping is hierarchical cluster analysis, which groups patients into an optimal number of clusters based on similarities in brain abnormality patterns. This is achieved using an iterative bottom-up approach. Patients begin as individual clusters that are merged stepwise according to a chosen linkage method, commonly *Ward’s method* (Ward, 1963) that tries to minimize total within-cluster variance and achieves well-separated, relatively equal-sized clusters that are less prone to outliers. Cluster merging is repeated until a single cluster remains. Clustering is therefore achieved at multiple hierarchical levels and visualized using a dendrogram to reveal the underlying data structure. Hierarchical cluster analysis does not require pre-defining the number of clusters based on hypothesized disease subtypes. Rather, the data-derived clusters can be compared based on imaging and non-imaging characteristics to infer meaningful disease subtypes.

Applying hierarchical cluster analysis to structural and diffusion MRI data, Inguanzo et al. (2021) identified three PD subtypes that differed in their pattern of grey and white matter changes and the severity of cognitive impairments. Cao and colleagues (Cao et al., 2022) used cluster analysis to identify two distinct PD subtypes from structural and functional MRI. One group showed diffuse reductions in grey matter volumes, decreased visual cortex activity, with more severe motor and cognitive impairment. The other group showed less brain atrophy, modest changes in frontotemporal and sensorimotor activity, and less severe motor and cognitive deficits. Classifying PD patients from the PPMI cohort, Maia et al. (2020) reported two subgroups: the first group was characterized by greater brainstem atrophy and an older age of onset, whereas the second group showed cortical and striatal atrophy and a younger age of onset.

Building on these traditional clustering approaches, partial least squares path modeling of cross-sectional imaging data has been proposed as a multivariate framework to infer different disease progression pathways within distinct subtypes. Pyatigorskaya et al. (2021) applied this model to structural, diffusion-weighted, and neuromelanin-sensitive MRI data from PD patients with and without REM sleep behavior disorder (RBD). They found that PD with RBD demonstrated a “brainstem-to-cortex” pattern of brain changes, whereas those without RBD followed the reverse “cortex-to-brainstem” trajectory. These findings appear to follow the proposed “brain-first/body-first dichotomy” hypothesis of PD progression (Horsager et al., 2020).

More recently, Subtype and Stage Inference (SuStaIn) has been introduced as an event-based method of disease stratification using clinical and imaging features (Young et al., 2018). This computational model uses ML to identify and characterize spatiotemporal disease subtypes from cross-sectional patient data spanning the disease timeline. The identified subtypes describe distinct patterns in the trajectory or sequence of biological abnormalities, which can reveal different pathological mechanisms or disease epicenters. Within each subtype, patients are also assigned a stage based on their cumulative degree of measured abnormality and this label describes where they are situated along the subtype-specific disease course. Like hierarchical cluster analysis, SuStaIn is entirely data-driven and does not rely on predefined models of disease progression subtypes.

Zhou et al. (2023) identified two distinct PD subtypes by applying SuStaIn to diffusion and neuromelanin-sensitive MRI of midbrain nuclei and limbic regions, as well as clinical assessments. They applied the model to a large discovery dataset (>200 PD, >100 HC) and validated their findings in a separate sample. One subtype was characterized by initial atrophy of the substantia nigra and locus coeruleus and early presentation of RBD and autonomic dysfunction. Degeneration of limbic regions and cognitive impairment followed at later stages. In contrast, the other subtype manifested early substantia nigra and limbic region atrophy, hyposmia, and cognitive impairment. This was followed by locus coeruleus changes and sleep disturbances in advanced disease. This accords with the brain-first/body-first dichotomy. Likewise, other studies using SuStaIn to model MRI data in PD have reported disease subtypes mainly distinguished based on the earliest affected regions, namely the cortex, subcortex, or brainstem (Sakato et al., 2024; Shawa et al., 2025). In sum, such studies in different well-sized samples point to the existence of consistent spatial progression subtypes.

Oxtoby and colleagues (Oxtoby et al., 2021) used event-based modeling to describe the clinical progression of PD patients from early changes in olfaction and sleep, initial deficits in visual cognition and increased brain iron content, before substantia nigra and cortical atrophy, retinal thinning, and eventual cognitive decline. Oh et al. (2024) found that a cohort of PD patients with normal cognition, mild cognitive impairment, and dementia could be classified into early, intermediate, and late disease stages, respectively. SuStaIn models of MRI and PET-derived dopamine availability revealed distinct spatiotemporal trajectories of subcortical atrophy and dopamine availability between these subgroups. Together, these studies showcase that data-driven disease progression models not only subtype PD patients based on spatially distinct brain patterns but also stage patients along clinically meaningful trajectories.

## 4 Pathophysiological modeling

Computational models can also be applied to imaging data to test hypotheses of the biological mechanisms underlying PD pathophysiology. One such hypothesis posits that PD is caused by the prion-like propagation of misfolded *α*-synuclein proteins from neuron to neuron (Jucker and Walker, 2013; Brundin and Melki, 2017). At the macroscopic level, this suggests that the disease targets and follows intrinsic brain networks (Seeley et al., 2009; Warren et al., 2013). To test this theory of network spread in human imaging data, we can generate disease models based on structural or functional connectomes from diffusion and functional MRI in healthy populations. These network models are based on graph theory, with brain regions represented by nodes and the connections between them represented as edges (Bassett and Sporns, 2017). Normative connectome properties can be derived and related to maps of observed brain changes measured in patients. Additionally, network models can be annotated with the patient-derived disease map to understand how connectome architecture shapes disease spread (Bazinet et al., 2023).

We applied network modeling to a large MRI dataset of PD to demonstrate the role of connectivity in disease spread. More specifically, we showed how atrophy within a given region is proportional to the atrophy of its connected neighboring regions. Regional atrophy in *de novo* PD was correlated with connectivity to substantia nigra (Zeighami et al., 2015), a presumed disease epicenter, and connectivity continues to explain the atrophy pattern in PD patients followed longitudinally (Tremblay et al., 2021). These findings based on deformation-based morphometry are mirrored by studies of cortical thinning in *de novo* PD (Yau et al., 2018; Vo et al., 2023). Network connectivity has also been reported to explain disease spread in prodromal PD (Rahayel et al., 2022) as well as cross-sectionally across disease stages (Vo et al., 2025), emphasizing its role spanning the PD course.

Network models spotlight global connectivity as the central driver of disease spread in PD. Local brain features, such as gene expression (Arnatkeviciute et al., 2021), cellular composition (Wei et al., 2018), or neuroreceptor profiles (Hansen et al., 2021), may also confer selective vulnerabilities to disease. However, these alternative mechanisms tend to correlate with intrinsic brain networks and could produce atrophy patterns that resemble network organization. Dynamic network models enriched with microstructural properties and set with kinetic system parameters might better capture the complex pathophysiology in PD.

Network diffusion models describe a spread of pathogenic proteins from a disease epicenter to connected brain regions via a diffusion process (Raj et al., 2012). Freeze et al. (2019) used a network diffusion model to estimate seed region probabilities from maps of MRI-derived atrophy in PD and expression of PD risk factor genes from the Allen Human Brain Atlas. They identified the substantia nigra and regions with high expression of microglial and immune-related genes as the most vulnerable disease epicenters. Building on this work, Pandya et al. (2019) showed a diffusion model of connectivity-based disease spread from the substantia nigra best explained the observed MRI-based atrophy pattern in PD. After integrating expression profiles of PD risk factor genes, they found both connectivity and–to a lesser extent–gene expression predicted the atrophy pattern.

To get at causal mechanisms, it may be useful to design biologically-informed disease propagation models. One such model is the Suseptible-Infected-Removed (SIR) agent-based model, which considers terms for both the propagation and clearance of pathogenic proteins (or agents) through a network (Iturria-Medina et al., 2014). We developed an agent-based SIR model that simulates the spread of *α*-synuclein proteins in the brain to explain the observed atrophy pattern in PD (Zheng et al., 2019). This model integrates global brain connectivity with local expression of *SNCA* and *GBA* genes that confer the regional synthesis and clearance of α-synuclein. Both baseline (Zheng et al., 2019) and longitudinal (Abdelgawad et al., 2023) atrophy patterns in PD could be successfully recreated. Importantly, null models that randomize connectivity and gene expression show near chance-level correlations with observed atrophy patterns in PD, supporting the role of both neural connectivity and α-synuclein synthesis and clearance in PD pathogenesis. Specifically, these findings suggest that regions with higher α-synuclein concentrations are more susceptible to atrophy and likely to propagate the disease along the connectome to neighboring regions.

## 5 Pitfalls and challenges of computational and ML models

Computational analysis of medical images holds promise for improving patient management. However, it faces significant challenges throughout the “research lifecycle” that hinder its clinical translation, from limitations in data quality and evaluation methodologies to publication biases (Varoquaux and Cheplygina, 2022). A recent meta-analysis reviewed neuroimaging-based studies using ML for PD diagnosis, prognosis, or intervention, systematically assessing their methodological quality using five minimal quality criteria (MQC), including data splitting, data leakage, model complexity, performance reporting, and indication of biological plausibility. Among the 244 studies within the scope, only 20% met all five MQC. The most common shortcomings included data leakage, non-minimal model complexity, and failure to report biological plausibility (Dzialas et al., 2024).

### 5.1 Data leakage/imbalance

Although computational experiments benefit from large, well-powered samples, size alone is not sufficient. To ensure clinically relevant predictions, test data must accurately represent the target population rather than being a random subset of the same data pool used for training. Dataset bias arises when the training data differ systematically from the data on which the model is ultimately applied. This mismatch can lead to overfitting, where algorithms perform well in benchmark evaluations but fail to generalize to real-world settings. Robust validation should go beyond single-dataset evaluation and aim for statistical consensus across multicenter datasets reflecting the diversity of imaging parameters, patient populations and disease heterogeneity. Additionally, class imbalance can further lead to overfitting and bias model performance (Varoquaux and Cheplygina, 2022; Dzialas et al., 2024).

Unbiased evaluation of model performance requires training and testing the models with independent datasets. Data leakage refers to the accidental sharing of information between the training and testing (or validation) datasets, giving the model access to information it would not have during real-world prediction. This leads to overly optimistic performance metrics and poor generalization. Separating all test data from the start, before any data transformation, helps avoid data leakage and ensures adequate performance evaluation (Varoquaux and Cheplygina, 2022; Dzialas et al., 2024). Based on the aforementioned meta-analysis, 16% of the studies did not address data imbalance, while only 7% validated their findings in external test sets, where the mean performance was significantly lower than that in test sets from the original dataset (Dzialas et al., 2024).

Labeling errors within datasets can also introduce biases that undermine the model performance and validity. A common example is diagnostic misclassification, which is due to the inherent limitations of current diagnostic criteria in Parkinsonian syndromes and the lack of pathological confirmation of diagnoses (Chougar et al., 2024).

### 5.2 Interpretability

On the other hand, explainable models are needed to interpret what is often seen as a “black-box,” enhance trust in predictions, and facilitate the clinical translation of AI-based tools. This includes reporting feature importance to understand pathophysiological plausibility and incorporating an individual-level analysis. Going beyond the global performance metrics, such analyses should focus on false positive and false negative subjects and explore the reasons behind their misclassification, which are overlooked in most studies (Chougar et al., 2024).

### 5.3 Publication bias

The current academic publishing paradigm may lead to pressure to publish only positive findings while underreporting negative results that may otherwise provide insights. As a result, critical methodological details may be omitted, undermining reproducibility. To address this, journal editorial boards should implement and enforce standardized ML quality criteria as part of the peer-review process (Varoquaux and Cheplygina, 2022; Dzialas et al., 2024). Notably, while the number of published studies has increased sharply over time, their methodological quality has not shown a corresponding improvement (Dzialas et al., 2024).

## 6 Conclusion

Machine learning techniques trained with imaging data have proven efficient in improving the diagnosis of Parkinsonism. Such approaches have the potential to aid clinicians in early-stage patient identification, particularly in non-expert centers. However, challenges remain to be addressed before these methods can be translated into clinical practice. Large-scale clinical trials will be crucial to assess their generalizability and their utility in the early stages when diagnostic uncertainty remains high. Beyond diagnosis, computational methods can also shed light on the pathophysiology of PD, leading to a better understanding of disease heterogeneity and facilitating patient stratification, ultimately promising improved therapeutic outcomes.
